# KI effects on corrosion inhibition for 1018 steel in acid media using *Medicago sativa*

**DOI:** 10.3389/fchem.2022.1032522

**Published:** 2022-11-10

**Authors:** A. Rodríguez-Torres, M. G. Valladares-Cisneros, A. Saldaña Heredia, J. G. González-Rodríguez

**Affiliations:** ^1^ Metropolitan Polytechnic University of Hidalgo, Tolcayuca, México; ^2^ School of Chemical Sciences and Engineering, Autonomous University of Morelos State, Cuernavaca, Morelos, México; ^3^ Research Center for Engineering and Applied Sciences, Autonomous University of Morelos State, Cuernavaca, Morelos, México

**Keywords:** *M. sativa*, corrosion inhibition, electrochemical techniques, efficiency, KI

## Abstract

*Medicago sativa (M. sativa)* extract is a safe and eco-friendly corrosion inhibitor of 1018 steel in acid media. The inhibitor reached a maximum efficiency of 85% by using 500 ppm. In this work, we study the use of KI as an add-on to increase the inhibition efficiency of *M. sativa*, as well as making the natural inhibitor competitive with the commercial ones. We evaluated the effect of halide ions through the variation of different concentrations of KI and its synergy with the extract of *M. sativa* as a corrosion inhibitor of carbon steel in 0.5 M sulfuric acid and at different temperatures. The results were obtained through electrochemical techniques such as electrochemical impedance spectroscopy (EIS), potentiodynamic polarization (PDP) curves, and weight loss gravimetric technique. It was found that halide ions increase the inhibition efficiency of *M. sativa* from 85 to 95% when 5 mM concentration of KI was used. The efficiency of the inhibition increases proportionally with the immersion time but reduces when the temperature increases. The addition of iodide ions (I−) revealed that it exerts a synergistic effect on the inhibition of corrosion with the extract of *M. sativa*. However, when studying the metal surface using a scanning electron microscope, pitting corrosion was found.

## Introduction

Steel is one of the few materials that have significance in human life. During the modern era, it drove architectural, technological, and even medical development, as it is a major constituent in buildings, bridges, vehicles, ships, and trains, among others.

Steel, compared to other materials of its type, has low production costs. The energy required for extracting iron from its ore is about 25% of what is needed for extracting aluminum. Steel is environmentally friendly as it can be recycled. Iron as an element is present in Earth’s crust by 5.6%, representing a secure raw material base (Gan 2011).

Carbon steels are used in a wide range of applications, such as structural components, industrial pipes, and kitchen appliances, and are considered a more economical option than the costly corrosion-resistant alloys. Carbon steels typically contain less than 1.5% carbon along with a low presence of Mn, Si, P, and S. Based on the percentage of carbon, the classification is further divided into three forms: low-carbon steels (<0.25% C), medium-carbon steels (0.25–0.70% C), and high-carbon steels (0.70–1.05% C) (Dwivedi et al., 2017).

The effects caused by corrosion on different metals are of much interest for industries, due to its impact on the economic loss and the failure of security systems, as they are exposed to aggressive media (Revie and Uhlig 2008). It has been looked for different alternatives for corrosion protection as there is preoccupation of having options with no effects on environment and following the standards, in which the use of green inhibitors is one of the most used alternatives.

The use of vegetal species as corrosion inhibitors has been widely used. However, in some cases, they have been reported to possess low efficiencies, and thus, they are not able to reach the standards for a good inhibitor. To increase the inhibitor efficiency, different research studies proved that the use of halide ions along with vegetal extracts as corrosion inhibitors reported higher efficiency. For example, *Occimum viridis* is used as a corrosion inhibitor for mild steel in sulfuric acid showing a maximum efficiency of 69%, which after adding 0.5 mM of KI showed an increase in the efficiency of 94.5% (Oguzie 2006). *Sansevieria trifasciata* as a corrosion inhibitor for aluminum in hydrochloric acid shows an initial efficiency of 87%, and by adding 0.5 mM of KI, it reaches a maximum efficiency of 94% (Oguzie 2007). Following this, in 2013, Priya conducted a study on *Abelmoschus esculentus* seeds and used as a corrosion inhibitor for mild steel in sulfuric acid where an efficiency of 94% was obtained. In this research, she proved different haloid salts such as KCl, KBr, and KI at 0.1 mM obtaining maximum efficiencies of 86, 91.5, and 99%, respectively, where KI is clearly the one that shows a better interaction with the extract and synergic parameter *S* with values ≥1 , which was attributed to its high hydrophobicity, a greater ionic radius, and low electronegativity of iodide ions (Priya et al., 2013; Hazazi et al., 2014).

A synergic effect of halides and quaternary ammonium salts on steel corrosion in sulfuric acid has been observed. The ion efficiency increases in the following order: Cl < Br < I. Pseudohalides also show synergism, while the fluoride ion does not show any synergic activity. Active anions are selectively chemisorbed on the metallic surface and raise the absorbability of the inhibitory cations, which interfere in the corrosion process (Räuchle F. and Díaz M. I. 1990).

*Medicago sativa (M. sativa)* extract has been previously proved as a corrosion inhibitor for 1018 steel in acid media showing a maximum efficiency of 90% after 8 h, and it maintained until 12 h. After this time, the inhibition efficiency decreases (Rodríguez Torres et al., 2016). The aim of the present work is to evaluate the effect of adding different concentrations of KI when using *M. sativa* as a corrosion inhibitor for 1018 steel in 0.5 M of H_2_SO_4_.

## Experimental procedure

### Testing metal

Steel samples with a chemical composition w/w%: C 0.14%, Mn 0.90%, P_max_ 0.05%, S_max_ 0.04%, and Fe± 98.87% were used. The samples were encapsulated in a commercial epoxy resin. It is used for corrosion protection covering almost the entire surface, leaving a defined contact area of 1 cm^2^ for electrochemical tests and 5.52 cm^2^ for gravimetric tests. Each of the samples was sanded with a carbide silicon paper from 100 to 600 grades to obtain a homogenous surface.

### Aggressive solution

Sulfuric acid was used at a reactive grade to prepare 0.5 M of H_2_SO_4_ solution.

### Inhibitory solution

To prepare the inhibitory solution, 500 ppm of *M. sativa* extract was used, as it was considered the best concentration as previously reported (Rodríguez Torres et al., 2016). We added different KI concentrations (1–5 mM) to evaluate the effect of halide ions on inhibition efficiency.

### Electrochemical evaluation

The electrochemical techniques used for the evaluation of *M. sativa* with different KI concentrations as a corrosion inhibitor were electrochemical impedance spectroscopy (EIS) and polarization potentiodynamic (PDP) curves. During the measurements, a conventional three-electrode cell was used, with 1018 carbon steel as a working electrode, Ag/AgCl as a reference electrode, and graphite as a counter electrode. PDP measurements were carried out with a speed rate of 1 mV/s at an interval of ±1000 mV from the corrosion potential. The corrosion current density values, *i*_corr_, were obtained using Tafel extrapolation. The inhibition efficiency (*η*) was calculated according to Eq. 1:

$$\eta \left(\%\right)=\left[\frac{i_{corr1}-i_{corr2}}{i_{corr1}}\right]*100,$$ (1)

where $i_{corr1}$ and $i_{corr2}$ are the current densities with and without an inhibitor, respectively.

EIS tests were carried out with a signal amplitude of 10 mV and a frequency interval between 0.05 and 10,000 Hz in an ACM instrument GillAC potentiostat.

## Results and discussion

### Electrochemical impedance spectroscopy

**FIGURE 1:**
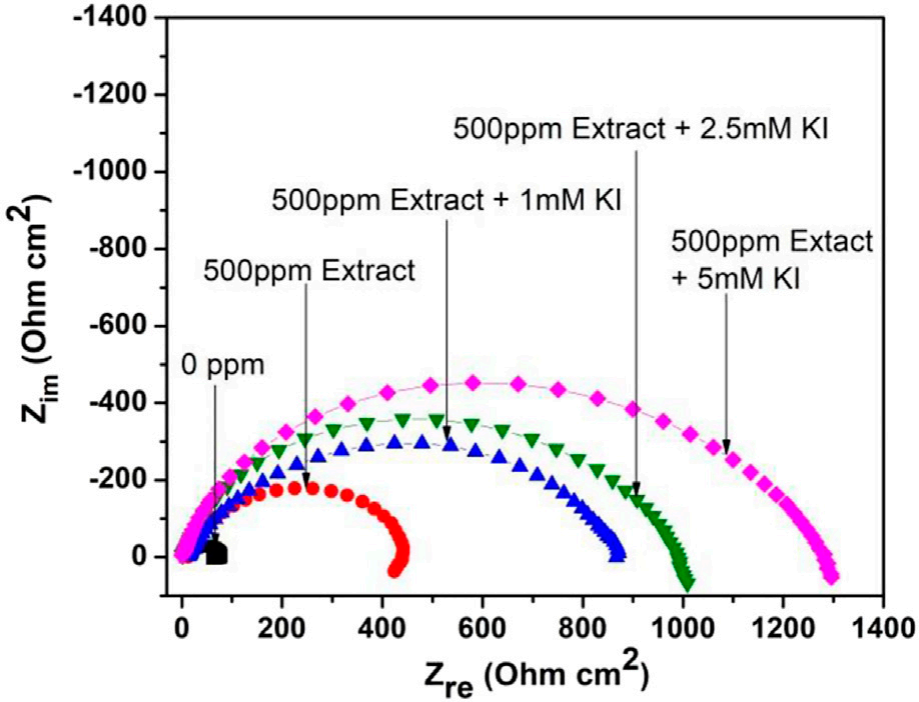
Nyquist diagram showing the effect of different concentrations of KI on the corrosion inhibition of 1018 steel in acid media using *M. sativa* aerial parts with 500 ppm concentration at 25 ± 2°C.

Figure 1 shows the Nyquist diagrams for the effect of different KI concentrations on 1018 steel corrosion inhibition in acid media using the aerial parts of *M. sativa* in a 500 ppm concentration. When KI is added, it shows flattened capacitive semicircles with the center in the real axis. This indicates that the corrosion mechanism is controlled due to the charge-transfer resistance (Bentiss et al., 2009; Behpour et al., 2010). By increasing the KI concentration in media, the diameter of the semicircles increases. This indicates an increase in the resistance and the produced efficiency in the interaction between halide ions and the inhibitor.

**FIGURE 2:**
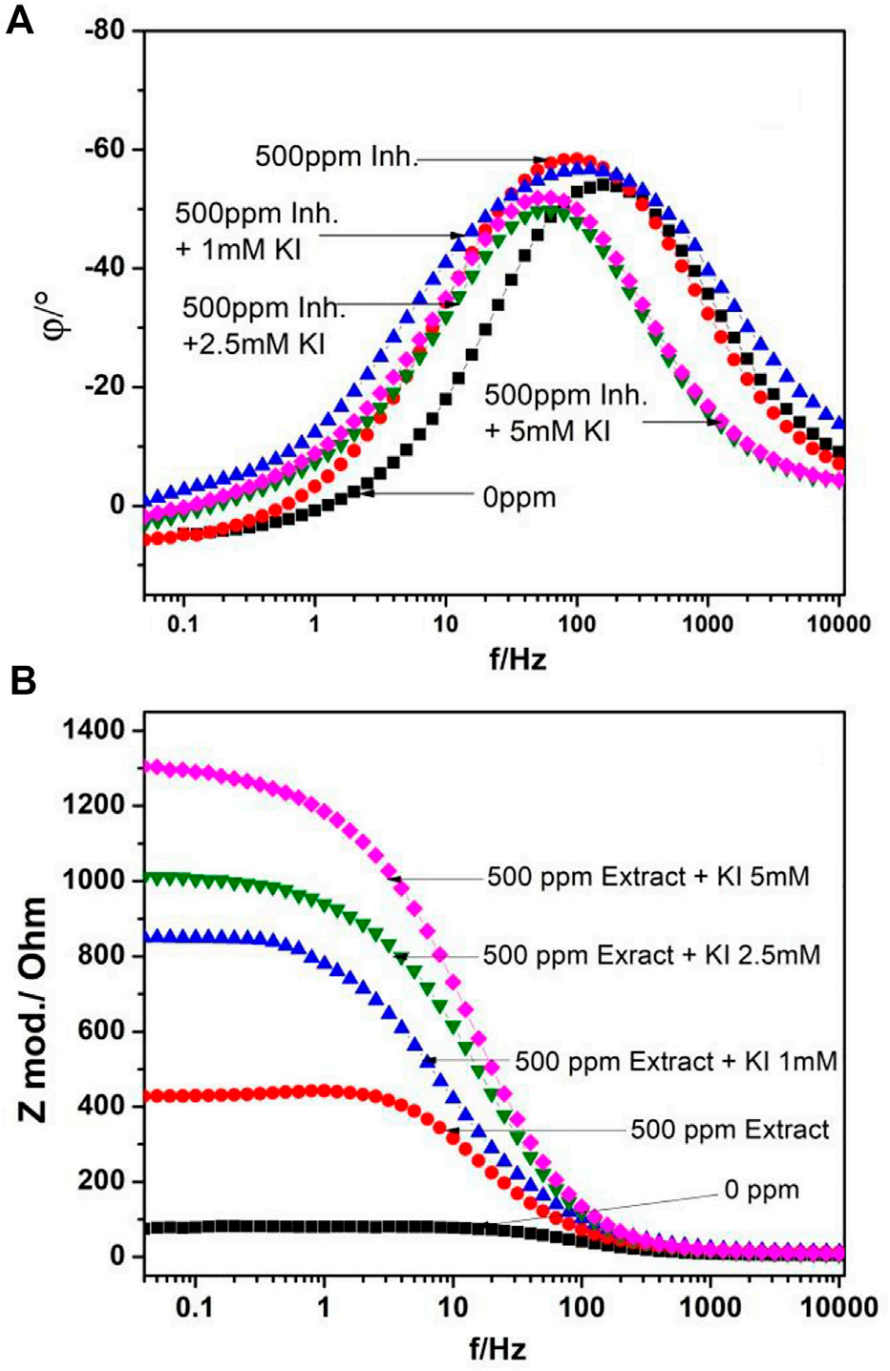
Bode diagrams for 1018 steel corrosion in acid media when adding 500 ppm of *M. sativa* at different KI concentrations **(A)** phase diagram and **(B)** module diagram.

Bode diagrams are shown in Figure 2. In phase diagram Figure 2A, a peak is observed around 100 Hz, which is displaced to lower frequencies as the KI concentration increases and the phase angle increases as the KI concentration increases. In module diagram Figure 2B, the solution resistance at high frequencies and at lower frequencies, the double-layer capacitance, and the charge-transfer resistance are shown, where the module value increases as the KI concentration increases, showing only one slope and as a consequence one time constant.

**FIGURE 3:**
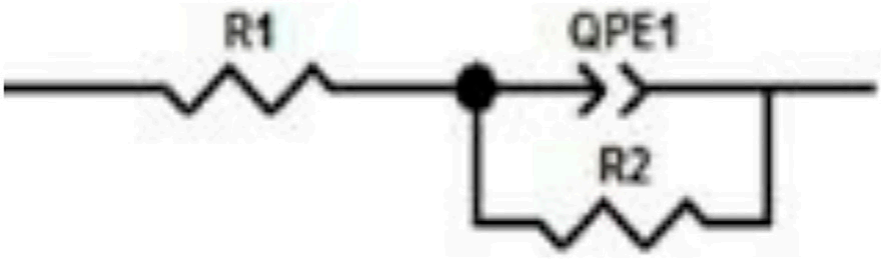
Adjusted and modeled data for the equivalent circuit of corrosion inhibition for 1018 steel in acid media with 500 ppm concentration of *M. sativa* and the addition of 5 mM of KI.

Figure 3 shows the equivalent circuit model, where R_1_ is the solution resistance and R_2_ is the charge-transfer resistance. The double-layer capacitance value is affected due to imperfections on the surface, such as roughness; thus, this effect is simulated through a constant phase element QPE1 (Bommersbach et al., 2006).

Impedance-associated values $Z_{CPE}$ can be calculated from the following equation (Tan et al., 2022; Singh et al., 2020):

$$Z_{CPE}=\frac{1}{Y_{0}{\left(j\omega \right)}^{n}},$$ (2)

where $Y_{0}$ is the pseudocapacitance, *j* is the current density, *ω* is the angular frequency, and *n* is a heterogeneity indicator or surface roughness. Depending on the *n* value, $Z_{CPE}$ can be represented as ( $Z_{CPE}=R$ , n=0 ), capacitance as ( $Z_{CPE}=C$ ; n=1 ), Warburg impedance as ( $Z_{CPE}=W$ , n=0.5 ), or inductance as ( $Z_{CPE}=L$ ; n=−1 ) (Aldana Gonzalez et al., 2015).

The double-layer capacitance value $C_{dl}$ can be calculated according to Eq. 3 (Tan et al., 2022; Bahremand et al., 2021):

$$C_{dl}=Y_{0}{\left(2\pi f_{z_{im-\max}}\right)}^{n-1}.$$ (3)

The obtained parameters for the simulation and efficiency calculus are shown in Table 1, where it is observed that as the KI concentration increases, the charge-transfer resistance increases and at the same time the inhibitor efficiency increases and the values of the double electrochemical layer decrease. This can be caused due to the local dielectric constant and/or due to the increase in thickness in the double electric (Kuznetsov and Andreev, 2014) layer. This suggests that the presence of the extract and halide ions modifies the double-layer electric structure due to the inhibitor molecules that act by adsorption in the metal/solution interphase (Cardozo da Rocha et al., 2010).

**TABLE 1:** Electrochemical parameters obtained from the model circuit and corrosion inhibition efficiency (IE) for 1018 steel in acid media with and without 500 ppm concentration of *M. sativa* at different KI concentrations.

Inh ppm	KI mM	R_S_ Ω cm^−2^	R_ct_ Ω cm^−2^	C_dl_ µF cm^−2^	C_PE_ µF cm^−2^	N	IE (%)	SD
0	0	6.7	58	4.30	9.40	0.91		
500	0	15.56	423	2.87	5.04	0.86	86	2.4
500	1	12.56	863	2.71	4.51	0.83	93	1.7
500	2.5	0.87	983	1.29	3.20	0.83	94	1.8
500	0.54	0.54	1,277	1.24	2.70	0.82	95	2.0

**FIGURE 4:**
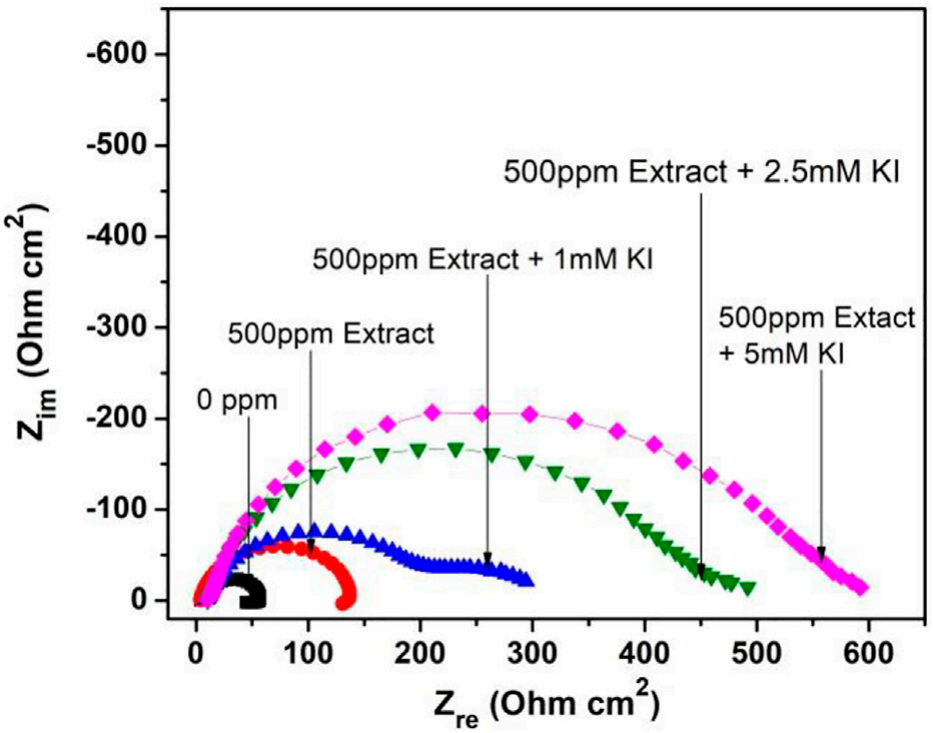
Nyquist diagram for 1018 steel corrosion in acid media with and without 500 ppm *M. sativa* extract and the addition of different KI concentrations at 40°C.

Temperature effect on the corrosion inhibition for 1018 steel in acid media at different KI concentration is shown in Figure 4. When different KI concentrations are added, the corrosion mechanism changes showing a depressed semicircle, followed by a second semicircle, which is more evident in 1 mM concentration. However, at high concentrations, it is not evident because the time constant is too low.

As a result, the corrosion process happens in two stages. The first is metal oxidation (charge-transfer process). The second stage is due to mass transport, as it is the slowest process where metallic ion diffusion could be exhibited from the metallic surface to the solution or the formation of a second corrosion product layer. Similar mechanisms have been observed for steel corrosion when using halide ions (Lagrenée et al., 2002).

**FIGURE 5:**
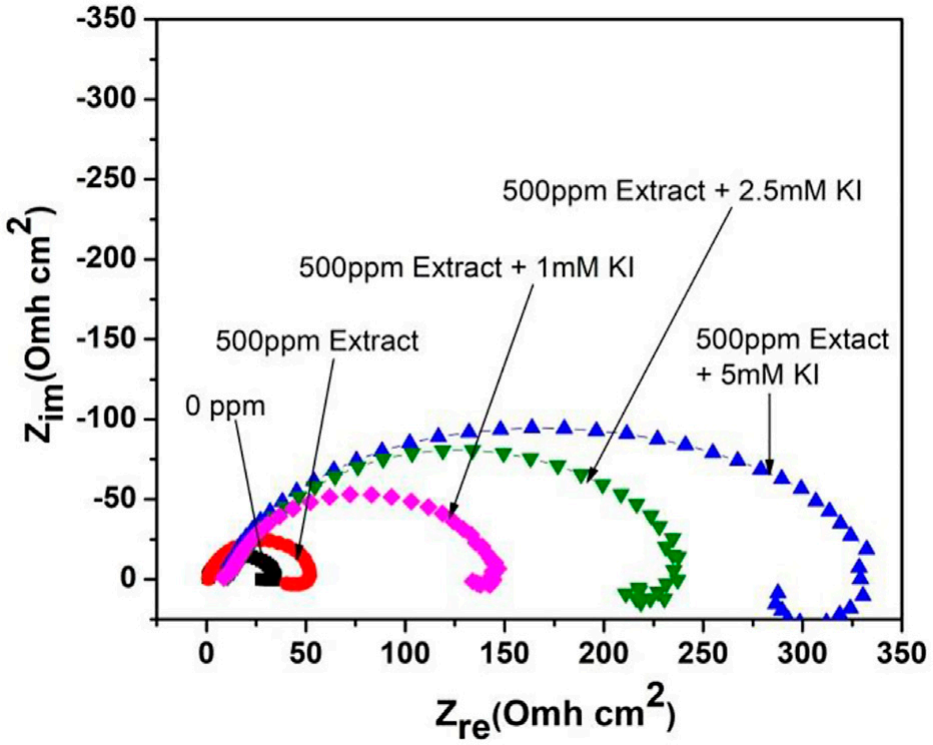
Nyquist diagrams for 1018 steel corrosion in acid media with and without 500 ppm of *M. sativa* and the addition of different KI concentrations at 60°C.

Figure 5 shows the effect of temperature when it is increased to 60°C for 1018 steel corrosion with 500 ppm *M. sativa* extract and different KI concentrations, and a capacitive behavior defining a depressed semicircle at high frequencies and an inductive semicircle at low frequencies are observed. As the KI concentration increases, the semicircle diameter increases, favoring *M. sativa* inhibition effect.

**FIGURE 6:**
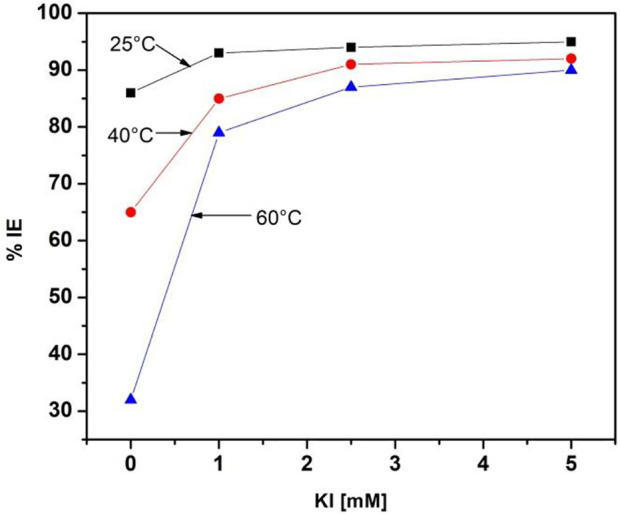
*M. sativa* inhibition efficiency comparison at 500 ppm with different KI concentrations and different temperatures.

Inhibition efficiency comparisons of 500 ppm *M. sativa* with different KI concentrations and different temperatures are shown in Figure 6. When the KI concentration increases, the efficiency increases considerably, and as the temperature increases, the efficiency decreases. When KI is added at a concentration of 5 mM, the efficiency values are maintained in the range of 91–95%.

**FIGURE 7:**
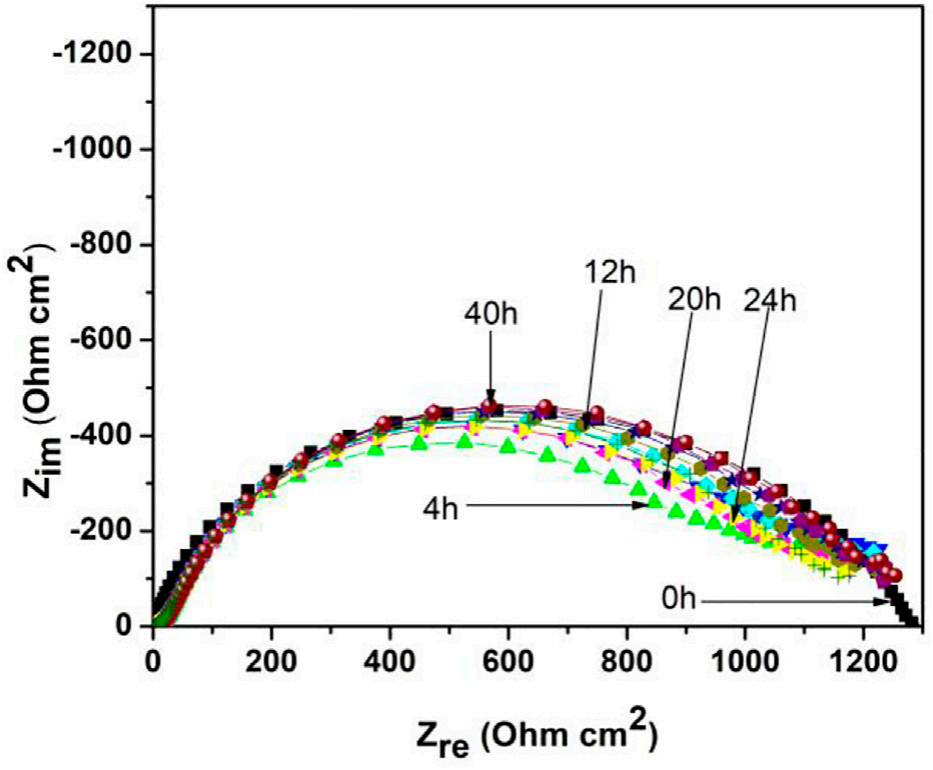
Nyquist diagrams at different exposure times of 1018 steel in acid media with 500 ppm of *M. sativa* and the addition of 5 mM KI at 25 ± 2°C.

Nyquist diagrams at different exposure times for 1018 steel in acid media with 500 ppm of *M. sativa* and the addition of 5 mM KI are shown in Figure 7. It is observed that the semicircle diameter remains almost constant for impedance values around 1,200 Ωcm^2^ until 40 h. However, its corrosion mechanism, at an initial time, is controlled by the charge-transfer resistance, with changes in time showing that for low frequencies, a second semicircle changing its mechanism into a two stage is observed; the first leading to metal oxidation (charge-transfer process) and the second stage is the mass-transfer process, thus causing a possible metallic ion diffusion from the metallic surface toward the solution or to the formation of a second layer.

This mass-transfer process is a characteristic of adsorption inhibitors due to the adsorption of their molecules onto the metal surface, which limits O_2_ diffusion on the surface or retains the metallic ions. This provokes a reduction in the corrosion rate (Feliu and Andrade 1991).

### Synergistic effect

The inhibition mechanism and the KI-adding effect require knowing the interaction between protective molecules and the metal surface. According to the steel dissolution mechanism in (Chauhan and Gunasekaran, 2007) sulfuric acid (Bockris et al., 1961), steel electrodissolution in an acid solution depends mainly on the adsorbed species as shown as follows:

$$Fe+H_{2}O↔\;{\left(FeOH\right)}_{ads}+H^{+}+e^{-}$$ (4)

$${\left(FeOH\right)}_{ads}\to \;{\left(FeOH\right)}^{+}+e^{-}$$ (5)

$${\left(FeOH\right)}^{+}+H^{+}\to {Fe}^{2+}+H_{2}O$$ (6)

The hydrogen evolution cathodic reaction is given by the following steps:

$$Inh-OH+H^{+}\;↔\;Inh-{OH}_{2}^{+}$$ (7)

$$Inh-COOH+{HSO_{4}}^{1-}\;↔\;Inh-{COO}^{-}+H_{2}SO_{4}$$ (8)

With the presence of halide ions (X), the anodic dissolution is given by:

$$\left[Fe{\left(H_{2}O\right)}_{n}{SO}_{4}\right]+I^{-}\to {\left[FeI{\left(OH\right)}_{n}SO_{4}\right]}_{ads}+H^{+}+e^{-}$$ (9)

$${\left[FeI{\left(OH\right)}_{n}SO_{4}\right]}_{ads}+InhNH_{3}^{+}\to [FeI{\left(OH\right)}_{n}SO_{4}InhNH_{3}{]}_{ads}^{+}$$ (10)

$$FeI{\left(OH\right)}_{n}SO_{4}InhNH_{3}{]}_{ads}^{+}\to {\left[FeI\;InhNH_{2}\right]}_{ads}+{OH}^{-}+SO_{4}^{2-}+H^{+}$$ (11)

Some aspects such as its polarization ease, high hydrophobicity, and its low electronegativity in comparison with other halides make the iodide ions have good chemisorption onto the metallic surface, favoring a better synergic inhibitor (Chin and Nobe, 1972; MacFarlane and Smedley, 1986; Jeyaprabha et al., 2006).

Another factor that promotes the adsorption of organic molecules is the chemisorption of I^−^ ions which is capable of decreasing the hydrophilicity of metal surfaces. However, this effect is much less significant than the surface charge effect (Kuznetsov 1996).

The inhibitor is adsorbed due to the metallic surface’s charge attraction (Priya et al., 2013), where iodide ions are being chemisorbed. This electrostatic interaction between the inhibitor and halide ions makes a greater surface coverage and therefore an increase in its efficiency (Umoren et al., 2010; Azim et al., 1995).

On the other hand, the cathodic reaction with an inhibitor is given as follows:

$${\left(FeH_{3}O^{+}r_{)}+I^{-}\to (rFeIH_{3}O\right)}_{ads}$$ (12)

$${\left(FeIH_{3}Or_{)}+InhNH_{3}^{+}\to (rFeI\;InhNH_{2}\right)}_{ads}+H_{2}O+H_{2}$$ (13)

The synergism parameter (S) between the inhibitor and the halide ions was calculated using the Aramaki and Hackermann relation (Obot et al., 2010; Oguzie et al., 2004; Umoren and Ebenso 2007):

$$S=\frac{1-I_{1+2}}{1-I_{1+2}^{´}},$$ (14)

where $I_{1+2}$ is the sum of the individual efficiencies of the inhibitor and halide ions and $I_{1+2}^{´}$ is the combined efficiencies of the inhibitor and halide ions. In Table 2, the calculated values for the synergism parameter are shown. When the value is S > 1, it indicates that there is a positive synergic effect, and when S < 1, it prevails an antagonist interaction due to a competitive adsorption and the halide ions (Obot et al., 2011).

**TABLE 2:** Synergism parameter calculated for 500 ppm of *M. sativa* and the 5 mM KI system.

Parameter	System Inh./KI	Temperature (°C)		
		25	40	60
IE (%)	0 ppm, 5 mM	62	58	39
	500 ppm, 0 mM	86	62	38
	500 ppm, 5 mM	95	92	91
Synergic parameter (S)	500 ppm, 5 mM	1.56	1.31	1.00

The values at room temperature and 40°C were greater than 1, indicating the existence of a synergic effect that favors metal protection, while at 60°C, the synergic parameter is equal to 1, suggesting that the interaction between inhibitor compounds and halide ions has been lost, but not speeding up the corrosion process in metal.

### Potentiodynamic polarization (PDP) curves for the aerial parts of *M. sativa* extract

**FIGURE 8:**
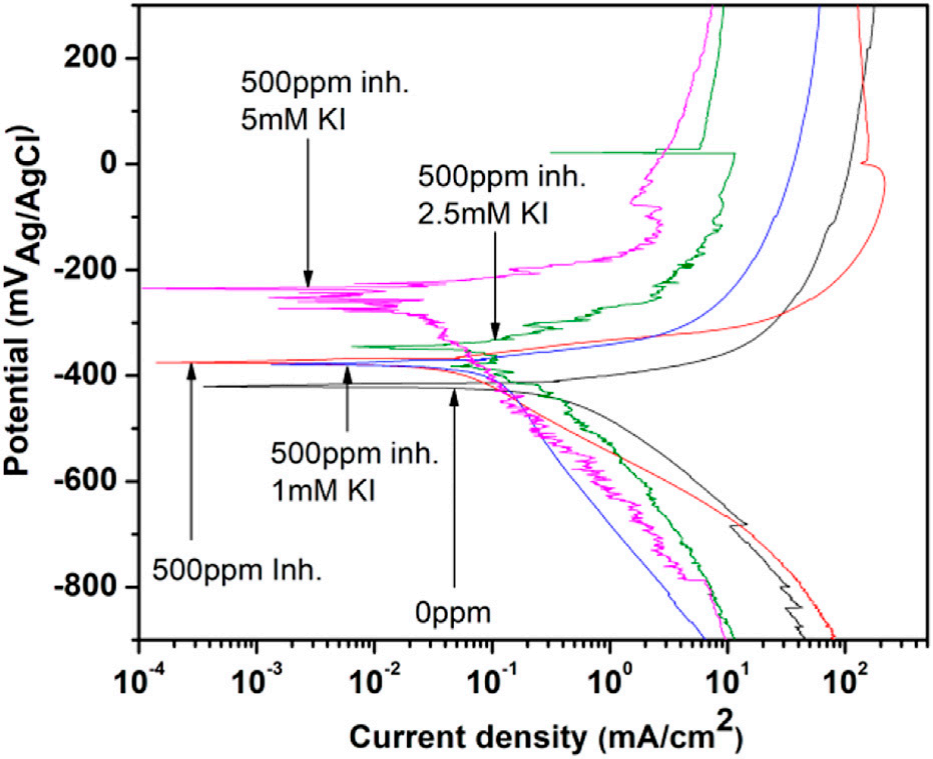
Polarization curves for 1018 steel corrosion inhibition with 500 ppm of *M. sativa* and different KI concentrations.

In Figure 8, the polarization curves for 1018 steel corrosion in acid media with 500 ppm of *M. sativa* at different KI concentrations are shown. An evident decrease in the corrosion density is observed, and at the same time, the corrosion potential is displaced to noble potentials when the KI concentration increases, exhibiting metal protection (Ahamad et al., 2010). During the test, when 2.5 and 5 mM of KI were added, a slight passivation around 22 and −86 mV, respectively, is observed.

Electrochemical parameters were obtained from the polarization curves and are shown in Table 3, where E_corr_ is the corrosion potential, log i_corr_ is the corrosion current density*, β*_a_ is the anodic slope, *β*_c_ is the cathodic slope, and IE is the inhibition efficiency associated to the corrosion current. It is observed that the corrosion potential becomes more positive as the KI concentration increases, indicating that the interaction between the extract and iodide ions acts in the anodic process (metal dissolution) (Yaro et al., 2011).

**TABLE 3:** Obtained values by Tafel extrapolation for 1018 steel corrosion inhibition with 500 ppm of *M. sativa* and different KI concentrations at 25 ± 2°C.

Inh ppm	KI mM	E_corr_ mV	i_corr_ mAcm^−2^	*β*_a_ mVdec^−1^	*β*_c_ mVdec^−1^	IE (%)	SD
0	0	−419	0.750	35	179	—	
500	0	−381	0.060	21	139	92	1.9
500	1	−379	0.080	39	282	89	2.1
500	2.5	−339	0.160	30	244	79	1.6
500	5	−241	0.023	34	238	97	1.2

However, the cathodic slope values increase. Meanwhile, the anodic slope values vary minimally with the presence of KI, showing that the corrosion process really affects the cathodic process (Chauhan and Gunasekaran, 2007).

The corrosion current density increases initially when adding 1 and 2.5 mM of KI; thus, the efficiency values decrease (Rodríguez Torres et al., 2016). However, by increasing the KI concentration to 5 mM, the current density decreases and the efficiency rises to 97%.

**FIGURE 9:**
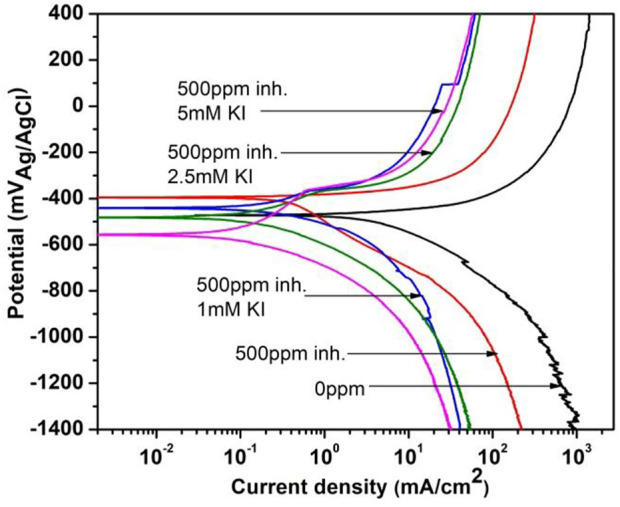
Polarization curves for corrosion inhibition of 1018 steel with and without 500 ppm of *M. sativa* and different KI concentrations at 40°C.

Figure 9 shows the polarization curves for corrosion inhibition of 1018 steel in acid media at 40°C, when 500 ppm of *M. sativa* and different concentrations of KI are added, where the current density decreases and the corrosion potential is displaced to more negative values when the KI concentration increases. Low passivation around −397 mV for 2.5 and 5 mM KI concentrations is observed.

Electrochemical parameters obtained from the polarization curves are shown in Table 4, where it is evident that by adding KI concentration, efficiency values increase. There is a low variation in cathodic and anodic slopes, which indicates that the inhibition process affects both reactions during the corrosion process (Hazwan Hussin and Jain Kassim, 2011).

**TABLE 4:** Electrochemical parameters obtained from the polarization curves for corrosion inhibition of 1018 steel in acid media with 500 ppm of *M. sativa* and different KI concentrations at 40°C.

C_Inh._ (ppm)	KI (mM)	E_corr_ mV	i_corr_ mAcm^−2^	*β*_a_ mVdec^−1^	*β*_c_ mVdec^−1^	IE (%)	SD
0	0	−474	4.30	70	249	—	
500	0	−401	0.36	28	158	91	1.7
500	1	−440	0.18	77	89	96	2.2
500	2.5	−481	0.15	85	142	96	1.8
500	5	−557	0.14	92	156	97	2.1

**FIGURE 10:**
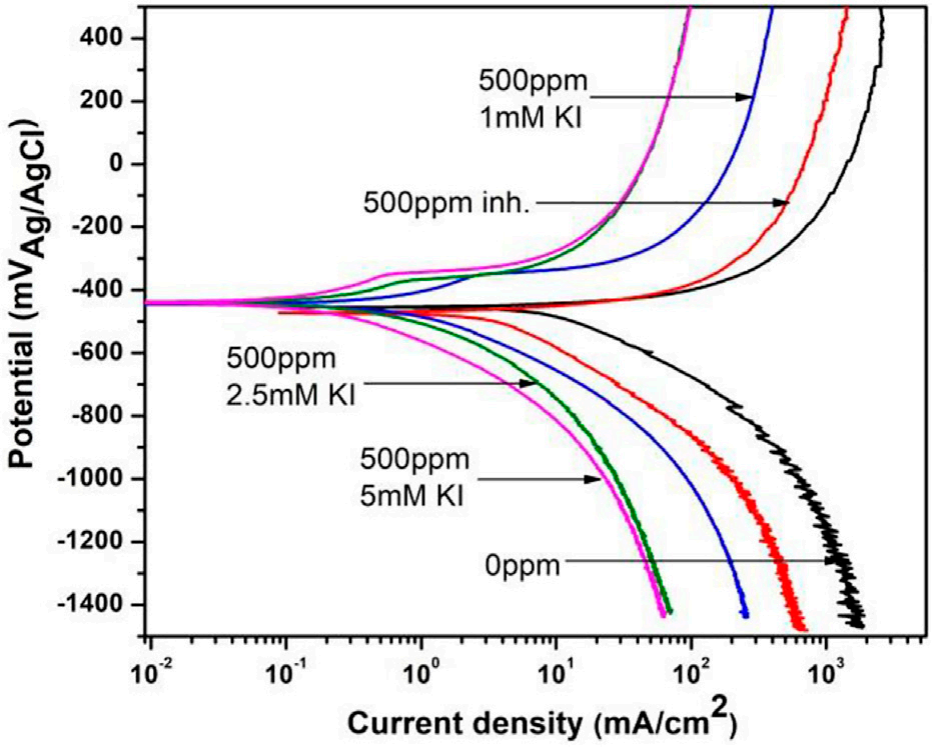
Polarization curves for corrosion inhibition of 1018 steel with and without 500 ppm of *M. sativa* and different KI concentrations at 60°C.

The polarization curves for 1018 steel corrosion with and without 500 ppm of *M. sativa* and different KI concentrations at 60°C are shown in Figure 10. It is seen that the corrosion current density is displaced to lower values according to the curve with no inhibitor and with no KI and few passivation around −350 mV for curves when KI was added.

Electrochemical parameters for these polarization curves are shown in Table 5. There are no significant variations in the corrosion potential and anodic and cathodic slopes. However, the corrosion current decreases and the efficiency increases from 21 to 97% as the KI concentration increases.

**TABLE 5:** Electrochemical parameters obtained from the polarization curves for corrosion inhibition of 1018 steel in acid media with 500 ppm of *M. sativa* and different KI concentrations at 60°C.

C_inh_ ppm	KI mM	E_corr_ mV	i_corr_ mAcm^−2^	*β*_a_ mVdec^−1^	*β*_c_ mVdec^−1^	%IE	SD
0	0	−457	10.59	70	208	—	
500	0	−468	4.70	50	241	21	2.2
500	1	−443	0.87	73	200	92	1.7
500	2.5	−435	0.56	57	211	94	1.9
500	5	−441	0.28	60	221	97	2.3

### Weight loss gravimetric technique

**FIGURE 11:**
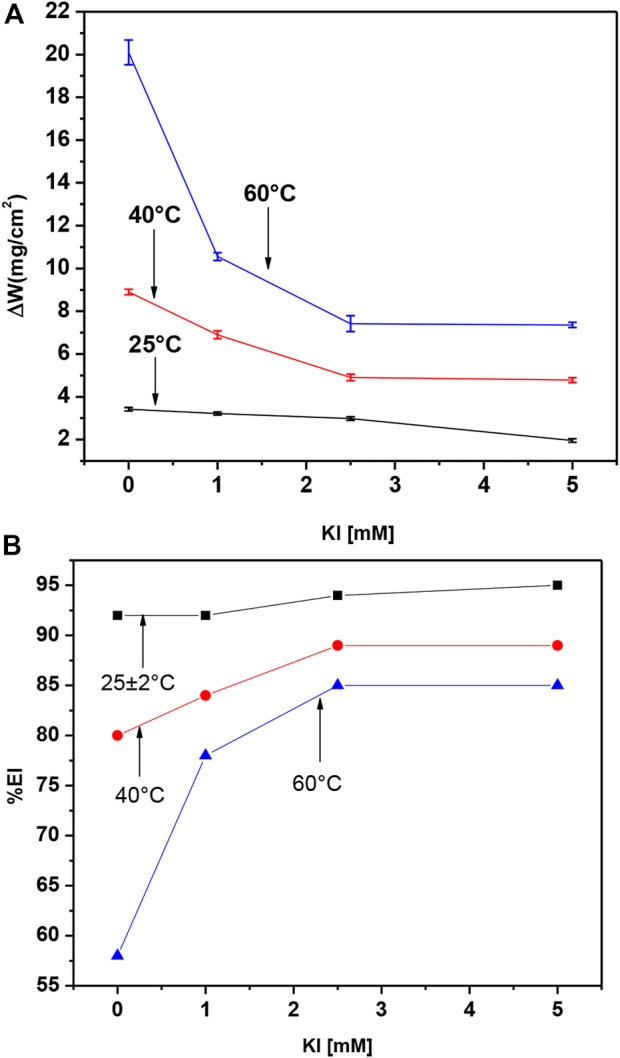
Effect of KI concentration and temperature on **(A)** weight loss and **(B)** inhibition efficiency for 1018 steel in acid media with 500 ppm of *M. sativa.*

For gravimetric tests, we selected 8 h *M. sativa* residence time to compare the inhibitor efficiency with and without KI addition. The obtained values for weight loss of 500 ppm *M. sativa* are shown in Figure 11A, proving that by increasing the KI concentration, the weight loss decreases. However, when the temperature increases, the weight loss increases too. It is shown in Figure 11B that by adding and increasing the KI concentration, the inhibitor efficiency increases, and then it decreases when the temperature increases. However, at 60°C, when 5 mM of KI is added, the efficiency is acceptable with 85%.

### Adsorption isotherm model analysis

In order to understand the adsorption phenomena of *M. sativa* and KI molecules on the metal surface, the use of adsorption models are very common (Nwosu et al., 2013; Christov and Popova, 2004; Eddy and Ebenso, 2008). We used different adsorption isotherms to fit the weight loss results. These adsorption isotherms contain Langmuir, Freundlich, Temkin, El-Awady, and Flory–Huggins. From all the employed isotherms, the best fitting and description of the adsorption behavior of *M. sativa* with different KI concentrations was obtained with the Flory–Huggins adsorption isotherm, which is represented by:

$$\ln\frac{\theta }{C}=xln\left(1-\theta \right)+\ln\left(xK_{ads}\right),$$ (15)

where $K_{ads}$ represents adsorption at a desorption equilibrium constant, *C* is the concentration of *M. sativa* and KI, x is the size parameter, and *n* is a measure of the number of adsorbed water molecules substituted by given inhibitor molecules. The covered surface degree θ was calculated using the inhibition efficiency from weight loss gravimetric technique ${EI}_{W}$ as follows:

$$\theta =\frac{{EI}_{W}}{100}.$$ (16)

**FIGURE 12:**
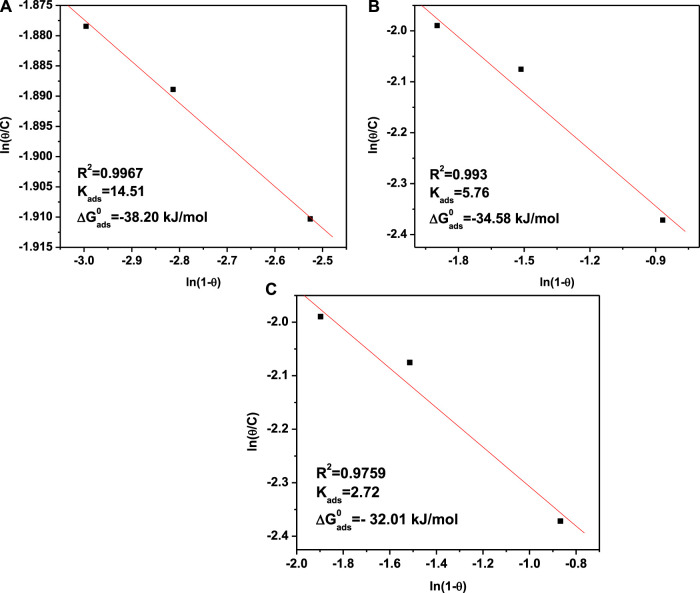
Flory–Huggins adsorption isotherms from 1018 carbon steel in 0.5 M H_2_SO_4_ with 500 ppm *M. sativa* and different KI concentrations at **(A)** 25°C, **(B)** 40°C, and **(C)** 60°C.

Figure 12 shows the dependence of ln (θ/C) vs. ln (1-θ), where a good fitting process can be observed. The adsorption coefficient *R* ^2^ of *M. sativa* and KI in the interface Fe/solution is consistent with (Hazazi et al., 2014) the Flory–Huggins adsorption isotherm (Nwosu et al., 2013).

In order to confirm the interaction between the inhibition concentration and the steel surface, the standard free adsorption energy ${∆G}_{ads}^{0}$ was calculated using the following equation:

$${∆G}_{ads}^{0}=-2.303RT\;\ln\left(55.5K\right),$$ (17)

where *R* is the gas constant, 55.5 is the molar concentration of water in the solution, and *T* is the absolute temperature.

$∆G_{ads}^{0}$ values can indicate the inhibitor adsorption type onto the metal surface. Small values [around -20.0 (Umoren and Ebenso, 2007) KJmol^-1^ or less negative] indicate a physical adsorption process, where the attraction and repulsion forces between the inhibitor and the metal surface prevail. On the other hand, $∆G_{ads}^{0}$ values around −40 kJ·mol^-1^ or higher indicate a chemical adsorption process due to the (Tan et al., 2022b) formation of coordination bonds between the inhibitor and the steel surface (Keleş et al., 2008). The values were obtained along −32.01 kJ·mol^-1^ ≥ $∆G_{ads}^{0}$ ≤−38.20 kJ·mol^-1^ at the evaluated temperatures, showing that the adsorption process of *M. sativa* extract with KI molecules is physical. Additionally, the value of $∆G_{ads}^{0}$ is negative, indicating that the adsorption of the inhibitor on the steel surface can proceed spontaneously (Tan et al., 2022).

### Metallic Surfaces analysis by Scanning Electron Microscope (SEM)

**FIGURE 13:**
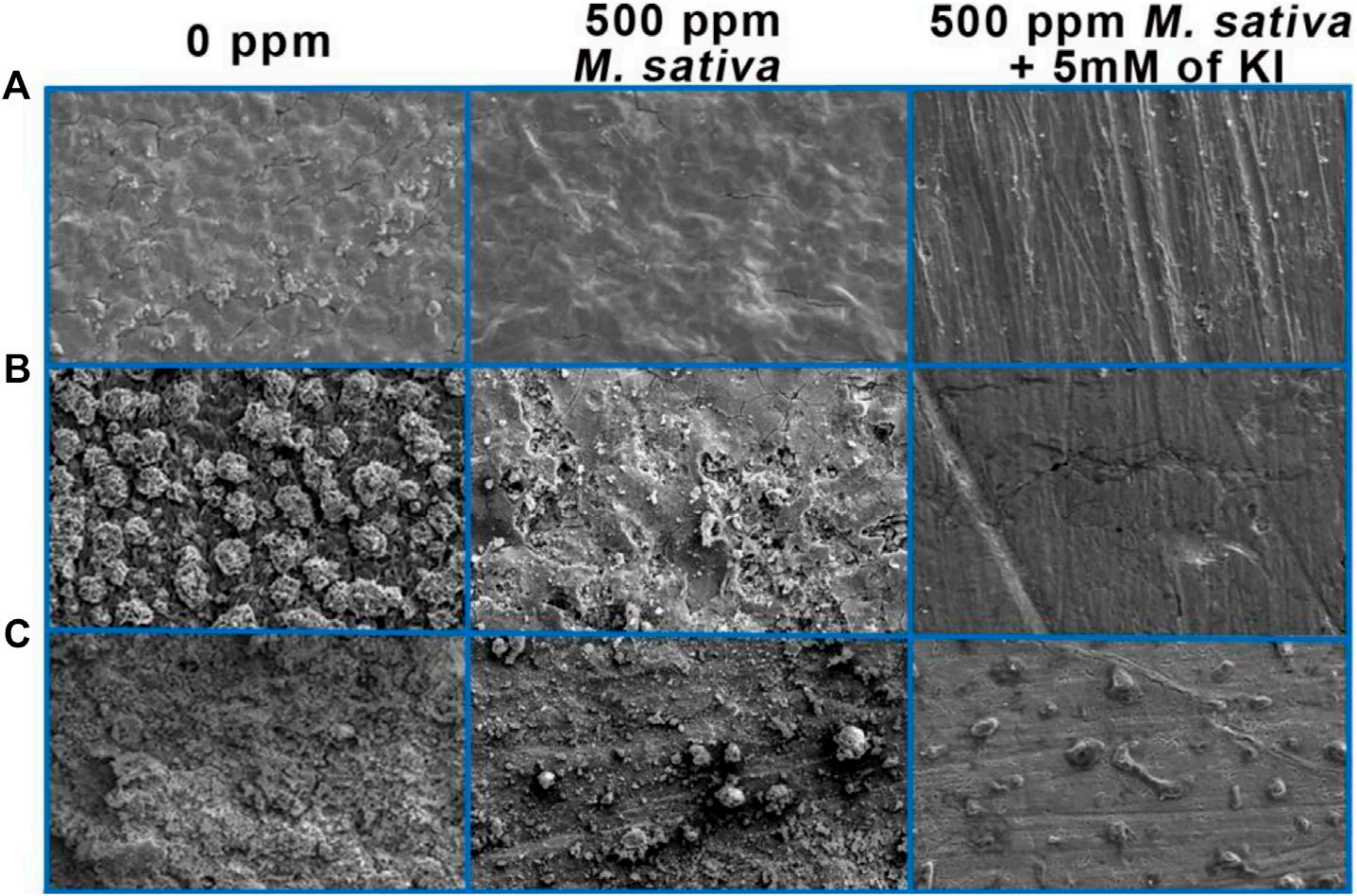
Micrographs of 1018 steel after exposure to acid media with and without 500 ppm *M. sativa* and 5 mM of KI at different temperatures. **(A)** 25°C ± 2°C, **(B)** 40°C, and **(C)** 60°C.

Surface analysis comparative with and with no 500 ppm of *M. sativa* and 5 mM of KI are shown in Figure 13. In the tests at 25°C ± 2°C (Figure 13A) is evident that when 5 mM of KI is added, metal surface is less damaged and cracking is not present by comparing with the micrograph with no inhibitor and when inhibitor is added. However by increasing the temperature to 40°C (Figure 13B) when KI is added, it can be observed pitting corrosion, this type of corrosion is one of the most dangerous due to metal structure sensitize provoking failures in equipment (Pourbaix, 1970; Groysman, 2010; Sarver and Edwards, 2012). At 60°C (Figure 13C) is shown a less damaged surface with the appearance of some corrosion products, pitting corrosion is not present however corrosion products could be covering the pits generated and hiding them in the micrograph.

## Conclusion

The data of electrochemical measurements indicate that *M. sativa* with different KI concentrations effectually inhibit the corrosion of 1018 steel in H_2_SO_4_ media. Additionally, the inhibition efficiency increases from 85 to 95% by adding 5 mM of KI.

The adsorption of *M. sativa* and KI at the steel interface is according to Flory–Huggins adsorption. Also, the adsorption of inhibitor molecules on to the steel surface is mainly through physical adsorption.

The inhibition efficiency decreases by raising the temperature. However, even at 60°C, the efficiency remains at 91%.

The addition of iodide ions (I^−^) revealed that it exerts a synergistic effect on the corrosion inhibition with the extract of *M. sativa*. However, when temperature increases, the metal surface becomes susceptible to pitting.

## Data Availability

The original contributions presented in the study are included in the article/Supplementary Material. Further inquiries can be directed to the corresponding author.

## References

[B1] Ahamad I. Prasad R. Quraishi M. A. (2010). Adsorption and inhibitive properties of some new Mannich bases of Isatin derivatives on corrosion of mild steel in acidic media. Corros. Sci. 52 (4), 1472–1481. 10.1016/j.corsci.2010.01.015

[B2] Aldana Gonzalez J. Espinoza Vazquez A. Romero Romo M. Uruchurtu Chavarin J. Palomar Pardave M. (2015). Electrochemical evaluation of cephalothin as corrosion inhibitor for API 5L X52 steel immersed in an acid medium. Arab. J. Chem. 12, 3244–3253. 10.1016/j.arabjc.2015.08.033

[B3] Azim S. S. Muralidharan S. Iyer V. (1995). Studies on the influence of iodide ions on the synergistic inhibition of the corrosion of mild steel in acidic solution. J. Appl. Electrochem 25, 495–500.

[B4] Bahremand F. Shahrabi T. Ramezanzadeh B. (2021). Development of a nanostructured film based on samarium (III)/polydopamine on the steel surface with superior anticorrosion and water-repellency properties. J. Colloid Interface Sci. 582, 342–352. 10.1016/j.jcis.2020.08.039 32827959

[B5] Behpour M. Ghoreishi S. M. Mohammadi N. Soltani N. Salavati-Niasari M. (2010). Investigation of some Schiff base compounds containing disulfide bond as HCl corrosion inhibitors for mild steel. Corros. Sci. 52, 4046–4057. 10.1016/j.corsci.2010.08.020

[B6] Bentiss F. Jama C. Mernari B. El-Attrari H. El-Kadi L. Lebrini M. (2009). Corrosion control of mild steel using 3, 5-bis(4-methoxyphenyl)-4-amino-1, 2, 4-triazole in normal hydrochloric acid medium. Corros. Sci. 51 (8), 1628–1635. 10.1016/j.corsci.2009.04.009

[B7] Bockris J. O’M. Drazic D. Despic A. R. (1961). The electrode kinetics of the deposition and dissolution of iron. Electrochim. Acta 4, 325–361. 10.1016/0013-4686(61)80026-1

[B8] Bommersbach P. Alemany-Dumont C. Millet J. P. Normand B. (2006). Hydrodynamic effect on the behavior of a corrosion inhibitor film: Characterization by electrochemical impedance spectroscopy. Electrochim. Acta 51, 4011–4018.

[B9] Cardozo da Rocha J. da Cunha Ponciano Gomesa J. A. D’Eliab E. (2010). Corrosion inhibition of carbon steel in hydrochloric acid solution by fruit peel aqueous extracts. Corros. Sci. 52, 2341–2348. 10.1016/j.corsci.2010.03.033

[B10] Chauhan L. R. Gunasekaran G. (2007). Corrosion inhibition of mild steel by plant extract in dilute HCl medium. Corros. Sci. 40, 1143–1161. 10.1016/j.corsci.2006.08.012

[B11] Chin R. J. Nobe K. (1972). Electrodissolution kinetics of iron in chloride solutions. J. Electrochem. Soc. 119, 1457–1461. 10.1149/1.2404023

[B12] Christov M. Popova A. (2004). Adsorption characteristics of corrosion inhibitors from corrosion rate measurements. Corros. Sci. 46, 1613–1620. 10.1016/j.corsci.2003.10.013

[B13] Dwivedi D. Lepková K. Becker T. (2017). Carbon steel corrosion: A review of key surface properties and characterization methods. RSC Adv. 7, 4580–4610. 10.1039/c6ra25094g PMC5378249 28413351

[B14] Eddy N. O. Ebenso E. E. (2008). Corrosion inhibitive properties and adsorption behaviour of ethanol extract of Piper guinensis as a green corrosion inhibitor for mild steel in H_2_SO_4”_ . Afr. J. Pure Appl. Chem. 2 (6), 046–054.

[B15] Feliu S. Andrade M. C. (1991). Corrosión y protección metálicas (vol. I). Madrid: CSIC.

[B16] Gan Y. (2011). “Advanced steel and our society: Better steel, better world (opening address and the introduction of the specific proceedings),” in Advanced steels. Editors Weng Y. Dong H. Gan Y. (Berlin, Heidelberg: Springer). 10.1007/978-3-642-17665-4_1

[B17] Groysman A. (2010). Corrosion for everybody. Springer Sci., 55–56.

[B18] Hazazi O. A. Fawzy A. Awad M. (2014). Synergistic effect of halides on the corrosion inhibition of mild steel in H2SO4 by a triazole derivative: Kinetics and thermodynamic studies. Int. J. Electrochem. Sci. 9, 4086–4103.

[B19] Hazwan Hussin M. Jain Kassim M. (2011). The corrosion inhibition and adsorption behavior of Uncaria gambir extract on mild steel in 1 M HCl. Mat. Chem. Phys. 125 (3), 461–468. 10.1016/j.matchemphys.2010.10.032

[B20] Jeyaprabha C. Sathiyanarayanan S. Muralidharan S. Venkatachari G. (2006). Corrosion inhibition of iron in 0.5 molL−1 H2SO4 by halide ions. J. Braz. Chem. Soc. 17, 61–67. 10.1590/s0103-50532006000100009

[B21] Keleş H. Keleş M. Dehri İ. Serindağ O. (2008). Adsorption and inhibitive properties of aminobiphenyl and its schiff base on mild steel corrosion in 0.5 M HCl medium. Colloids Surfaces, A Physicochem. Eng. Aspects 320, 138–145. 10.1016/j.colsurfa.2008.01.040

[B43] Kuznetsov Y. I. (1996). Organic inhibitors of corrosion metals. United States: Plenum.

[B22] Kuznetsov I. Andreev N. N. (2014). Corrosion 96. Houston: NACE International.

[B23] Lagrenée M. Mernari B. Bouanis M. Traisnel M. Bentiss F. (2002). Study of the mechanism and inhibiting efficiency of 3, 5-bis(4-methylthiophenyl)-4H-1, 2, 4-triazole on mild steel corrosion in acidic media. Corros. Sci. 44, 573–588. 10.1016/s0010-938x(01)00075-0

[B24] MacFarlane D. R. Smedley S. I. (1986). The dissolution mechanism of iron in chloride solutions. J. Electrochem. Soc. 133, 2240–2244. 10.1149/1.2108381

[B25] Nwosu F. O. Nnanna L. A. Osarolube O. (2013). The use of eco-friendly leaf as a corrosion inhibitor of mild steel in an acidic environment. Int. J. Mater Chem. 3, 64–68.

[B26] Obot I. B. Obi-Egbedi N. O. Umoren S. A. Ebenso E. E. (2010). Synergistic and antagonistic effects of anions and Ipomoea invulcrata as green corrosion inhibitor for aluminium dissolution in acidic medium. Int. J. Electrochem. Sci. 5, 994–1007.

[B27] Obot I. B. Umoren S. A. Obi-Egbedi N. O. (2011). Corrosion inhibition and adsorption behaviour for aluminuim by extract of Aningeria robusta in HCl solution: Synergistic effect of iodide ions. J. Mat. Environ. Sci. 2 (1), 60–71.

[B28] Oguzie E. E. (2007). Corrosion inhibition of aluminium in acidic and alkaline media by *Sansevieria trifasciata* extract. Corros. Sci. 49, 1527–1539. 10.1016/j.corsci.2006.08.009

[B29] Oguzie E. E. Okolue B. N. Ebenso E. E. Onuoha G. N. Onuchukwu A. I. (2004). Evaluation of the inhibitory effect of methylene blue dye on the corrosion of aluminium in hydrochloric acid. Mat. Chem. Phys. 87, 394–401. 10.1016/j.matchemphys.2004.06.003

[B30] Oguzie E. E. (2006). Studies on the inhibitive effect of *Occimum viridis* extract on the acid corrosion of mild steel. Mater. Chem. Phys. 99, 441–446. 10.1016/j.matchemphys.2005.11.018

[B31] Pourbaix M. (1970). Significance of protection potential in pitting and intergranular corrosion. Corrosion 26 (10), 431–438. 10.5006/0010-9312-26.10.431

[B32] Priya V. S. Ali Fathima Sabirneeza A. Subhashini S. (2013). Synergistic effect of halides ions on the corrosion inhibition of *Abelmoschus esculentus* seed extract on mild steel in H2SO4. Asian J. Chem. 25 (13), 7083–7087. 10.14233/ajchem.2013.14440

[B33] Räuchle F. Díaz M. I. (1990). Inhibición de la corrosión. Rev. Quím. 4, 59.

[B34] Revie R. W. Uhlig H. H. (2008). Corrosion and corrosion control. An introdruction to corrosion science and engineering”. Canada: Wiley-Interscience.

[B35] Rodríguez Torres A. Valladares Cisneros M. G. González Rodríguez J. G. (2016). *Medicago sativa* as a green corrosion inhibitor for 1018 carbon steel in 0.5 M H_2_SO_4_ solution. Green Chem. Lett. Rev. 9 (3), 143–155. 10.1080/17518253.2016.1195017

[B36] Sarver E. Edwards M. (2012). Inhibition of copper pitting corrosion in aggressive potable waters. Int. J. Corros. 11, 1–16. 10.1155/2012/857823

[B37] Singh A. Ansari K. R. Chauhan D. S. Quraishi M. A. Lgaz H. Chung I. (2020). Comprehensive investigation of steel corrosion inhibition at macro/micro level by ecofriendly green corrosion inhibitor in 15% HCl medium. J. Colloid Interface Sci. 15, 225–236. 10.1016/j.jcis.2019.10.040 31670020

[B38] Tan B. Lan W. Zhang S. Deng H. Qiang Y. Fu A. (2022b). Passiflora edulia Sims leaves Extract as renewable and degradable inhibitor for copper in sulfuric acid solution. Colloids Surfaces A Physicochem. Eng. Aspects 645, 128892. 10.1016/j.colsurfa.2022.128892

[B39] Tan B. Zhang S. Cao X. Fu A. Guo L. Marzouki R. (2022). Insight into the anti-corrosion performance of two food flavors as eco-friendly and ultra-high performance inhibitors for copper in sulfuric acid medium. J. Colloid Interface Sci. 609, 838–851. 10.1016/j.jcis.2021.11.085 34838315

[B40] Umoren S. A. Ebenso E. E. (2007). The synergistic effect of polyacrylamide and iodide ions on the corrosion inhibition of mild steel in H2SO4. Mat. Chem. Phys. 106, 387–393. 10.1016/j.matchemphys.2007.06.018

[B41] Umoren S. A. Solomon M. M. Udosoro I. I. Udoh A. P. (2010). Synergistic and antagonistic effects between halide ions and carboxymethyl cellulose for the corrosion inhibition of mild steel in sulphuric acid solution. Cellulose 17, 635–648. 10.1007/s10570-010-9409-7

[B42] Yaro A. S. Khadom A. A. Ibraheem H. F. (2011). Peach juice as an anti-corrosion inhibitor of mild steel. Anti-Corrosion Methods Mater. 58 (3), 116–124. 10.1108/00035591111130497

